# Supplementary material for: Combined Metabolic Activators Accelerates Recovery in Mild‐to‐Moderate COVID‐19
Source: Adv Sci (Weinh). 2021 Jun 28;8(17):2101222. doi: 10.1002/advs.202101222 (PMC8420376; doi:10.1002/advs.202101222)
Supplement: Supplementary file 1 — Supporting Information [file ADVS-8-2101222-s013.pdf]

© 2021 Wiley-VCH GmbH

ADVANCED SCIENCE
Open Access

Supporting Information

for *Adv. Sci.,* DOI: 10.1002/advs.202101222

Combined Metabolic Activators accelerates recovery in mild-to-moderate COVID-19

*Ozlem Altay, Muhammad Arif, Xiangyu Li, Hong Yang, Mehtap Aydın, Gizem Alkurt,Woonghee Kim, Dogukan Akyol, Cheng Zhang, Gizem Dinler-Doganay, Hasan Turkez, Saeed Shoaie, Jens Nielsen, Jan Borén, Oktay Olmuscelik, Levent Doganay, Mathias Uhlén, and Adil Mardinoglu**

Supplementary Appendix

This appendix has been provided by the authors to give readers additional information about their work.

Supplement to: Combined metabolic activators accelerate recovery in mild-to-moderate COVID-19

Table of Contents

Supplementary Appendix..1

Study protocol..2

Table S1. The use of N-acetyl-L-cysteine, Nicotinamide Riboside (Vitamin B3, Niacin) and L-carnitine used in previous human trials associated with viral diseases including COVID-19.....................................8

Table S3. Summary of Univariate Cox regression analysis of overall treatment duration in Phase 2........12

Table S4. Summary of Univariate Cox regression analysis of overall treatment duration in Phase 3........12

SUPPLEMENTARY TABLES 18

SUPPLEMENTARY DATASETS...19

Study protocol

Indication Coronavirus Disease 2019 (COVID-19)

Study agent Combined metabolic activators (CMAs) consisting of serine, L-carnitine tartrate, N-acetylcysteine, and nicotinamide riboside

Study phase Phase II and Phase III

Methodology

Phase II: Open label, randomized controlled design

Phase III: Double blinded, randomized controlled design

Treatment arm: At home-treatment with standard therapy + CMAs consisting of serine, L-carnitine tartrate, N-acetylcysteine, and nicotinamide riboside as compared to standard therapy +

placebo. Standard therapy includes only hydroxychloroquine in phase II and either hydroxychloroquine or favipiravir in phase III.

Primary objective

Studies are planned as Phase II and Phase III clinical drug research to be conducted in patients diagnosed with COVID-19. Patients will be ambulatory and after the diagnosis/confirmation of diagnosis, will be sent home with their treatment.

The primary objective is to assess the clinical efficacy of the combination of metabolic activators administration and standard therapy in COVID-19 patients.

For the primary purpose, the proportion of patients who were recovered during the course of disease until 14 days after the initial diagnosis of COVID-19 disease.

Secondary objectives

The secondary aim is to evaluate the safety and tolerability of combined metabolic activators administration and standard therapy combination.

The following secondary safety objectives have been identified both in phase II and phase III studies:

• Number / characteristics of adverse event (AE), Serious Adverse Event (SAE) and treatment discontinuation due to study drug from the beginning of the study to the end of the follow-up period

• Number / features of all changes in hematology parameters evaluated as AE from the beginning of the treatment to the end of the follow-up period.

• Number / characteristics of all changes in selected biochemical parameters evaluated as AE from the beginning of the treatment to the end of the follow-up period.

• The changes in vital signs (systolic and diastolic blood pressures, pulse, respiratory rate, body temperature, pulse oximetry values), baseline values, and the status of treatment and follow-up visits

Number of subjects

A total of 100 COVID-19 disease patients in phase II will be enrolled and randomized on a 3:1 basis to the combined metabolic activators administration + standard therapy or placebo + standard therapy in Turkey. Standard therapy includes only hydroxychloroquine treatment.

A total of 300 COVID-19 disease patients in phase III will be enrolled and randomized on a 3:1 basis to the combined metabolic activators administration + standard therapy or placebo + standard therapy in Turkey. Standard therapy includes either hydroxychloroquine or favipiravir treatment.

Main inclusion criteria

To be included in the study, patients should meet all the following criteria:

- Patients of both genders (females and males) over 18 years of age
- Written informed consent obtained from the subjects prior to any procedures related to the study.
- Understand all procedures to be applied within the scope of the study protocol
- Ambulatory patients with symptoms diagnosed with COVID-19 with real time PCR test result positivity in the last 24 hours
- Patients with stable clinical course and who could be treated on an ambulatory basis

Main exclusion criteria

Subjects cannot be included in the study if any of the following criteria is met:

- Patients who has partial oxygen saturation below 90% or with severe clinical status requiring admission to critical care unit
- Inability or unwillingness to give written informed consent
- Physician makes a decision that trial involvement is not in patients' best interest, or any condition that does not allow the protocol to be followed safely.
- Patients considered as inappropriate for study for any reason
- Active participation in another clinical study
- Uncontrolled Type 1 or type 2 diabetes
- Severe liver disease (e.g. Child Pugh score ≥ C, AST>5 times upper limit)
- Patients with known severe renal impairment (estimated glomerular filtration rate ≤30 mL/min/1.73 m2) or receiving continuous renal replacement therapy, hemodialysis, peritoneal dialysis
- Significant cardiovascular co-morbidity (i.e. heart failure)
- Patients with phenylketonuria (contraindicated for NAC)
- Known allergy for substances used in the study
- Alcohol consumption over 192 grams for men and 128 grams for women per week
- Pregnant or breastfeeding, or positive pregnancy test in a pre-dose examination
- Receipt of any experimental treatment for COVID-19 within the 30 days prior to the time of the screening evaluation.

Dosage and duration of therapy

Phase II study was planned as a parallel group, randomized and open label study. The study subjects in phase II will be randomized on a 3:1 basis to the combined metabolic activators administration+ standard therapy or placebo + standard therapy in Turkey. Standard therapy includes only hydroxychloroquine treatment. Standard therapy + placebo group will include 25 volunteers; the standard therapy + combined metabolic activators administration group will consist of 75 volunteers.

Phase III study was planned as a parallel group, randomized and double-blinded study. The study subjects in phase III will be randomized on a 3:1 basis to the combined metabolic activators administration+ standard therapy or placebo + standard therapy in Turkey. Standard therapy includes one of the hydroxychloroquine or favipiravir treatment. Standard therapy + placebo group will include 75 volunteers; the standard therapy + combined metabolic activators administration group will consist of 225 volunteers.

In both studies, after the volunteers sign the informed consent forms, the treatment will be administered as the first by the responsible investigator. All other treatments will be administered by the patient at home. Hydroxychloroquine treatment will be administered at an initial dose of 800 mg/day (2x400 mg oral) followed by 400 mg/day (2x200 mg oral) for a total of 5 days. The dosage of favipiravir will be 1600 mg orally twice daily for 1 day followed by 600 mg orally twice daily for 4 days.

The standard therapy + combined metabolic activators administration group will be applied the same dosage and duration of the standard therapy. Additionally, CMAs will be given for two weeks in doses indicated below (was given orally, twice/day; one dose in the morning, one dose after dinner):

**Drug**	**gr in one Dose**
L-Carnitine tartrate	3.73 g/dose
N-Acetylcysteine	2.55 g/dose
Nicotinamide riboside	1 g/dose
Serine	12.35 g/dose

The total treatment period for the standard therapy will be 5 days and administration of CMAs will be for 14 days. On the 14th day, patients will perform a last visit which will be the follow-up visit.

Investigational Products Investigational products will be produced as the test product and will be packed as individual dosages in identical HDPE plastic bottles of 60 mL with a screw cap. Test product will be in the form of powder and will be dissolved in 200 ml preferably cold water before use. Placebo treatment will include lactose only in identical HDPE plastic bottles of 60 mL with a screw cap.

Study design

The study comprises two visits:

Visit 1 (Screening; Day 0):

The subjects will get the informed consent form and oral information on the study protocol. Blood samples and will be taken to check out the eligibility to the study.

After clinical and physical examination blood samples will be obtained for routine analysis outlined in the study protocol. Chest tomography (CT) and virus load assessment by PCR will be performed. An ECG will also be performed. Plasma samples will be taken for clinical chemistry analysis.

Eligible study subjects will be invited to the clinic for randomization to the active treatment and standard therapy groups. They will be asked to take the first dose and be observed for development of side effects. Patients who can tolerate the study agents will start to take combined metabolic activators (i.e., 2 dosage daily just after breakfast and dinner) for two weeks.

After initial dosing at the hospital patients will be instructed to stay at home during their treatment and will be asked to attend to the investigational site if symptoms are worsened or diminished during the 14-day period.

Visit 2 (Week 2, Day 14):

Clinical and physical examination, adverse events recording, virus load assessment by PCR will be performed. Chest tomography will be performed. Plasma samples will be taken for clinical chemistry analysis.

After the visit 2, subjects will stop taking their study drugs.

Study duration

The active treatment duration will be 2 weeks for each subject and the total study duration is estimated as 6 months.

Efficacy evaluation

The primary efficacy evaluation will be on the percentage (proportion) of patients recovered during the treatment period for both arms in COVID-19 patients.

The primary objective is to assess the clinical efficacy of the combined metabolic activators administration and standard therapy in COVID-19 patients based on the proportion of recovery.

Safety evaluation

Safety will be assessed by monitoring of adverse events, physical examination, vital signs measurements and clinical laboratory tests.

	**Visit**

**Visits**	**Visit 1**	**Visit 2**
**Days**	**Day 0**	**Day 14**
Informed consent	X	
Demographic data	X	
Surgical & medical history	X	X
Physical examination[1] and diagnosis	X	X
Inclusion/Exclusion criteria	X	
**Laboratory tests**		
Blood sample collection[2]	X	X
Chest tomography	X	X
ECG	X	
Virus load assessment by PCR	X	X
**Efficacy and safety evaluation**		
Laboratory safety parameters [3]	X	X
**Study drug administration**		
Randomization [4]	X	
Drug administration[5,6]	X	
Monitoring of compliance	X	X
Prior & concomitant medication	X	X
AE and SAE [7]	X	X

1. Physical examination will include body weight, height, body mass index, vital signs measurements.
2. At both visits, blood samples will be collected for laboratory tests.
3. Laboratory safety parameters will include complete blood count, alanine aminotransferase, aspartate aminotransferase, creatinine, C-reactive protein, triglycerides, cholesterol, glucose, LDH, ferritin, D-dimer.
4. Eligible study subjects at Visit 1 will be offered to be enrolled in the study and to take the first dose at the hospital.
5. Two doses taken just after breakfast and dinner. Patients will be treated on an ambulatory basis. If patients’ symptoms worsen, patient will be instructed to attend the hospital for an examination.

6. The study participants will be observed for the development of any allergic reactions or intolerance after taking the first dose at the hospital.
7. Adverse events (AE) and serious adverse events (SEA) will be monitored continuously and all AEs that occur at any time during the study will be reported in Case Report Forms.

Table S1. The use of N-acetyl-L-cysteine, Nicotinamide Riboside (Vitamin B3, Niacin) and L-carnitine used in previous human trials associated with viral diseases including COVID-19.

NCT Number	Title	Status	Interventions	Age (Years)	Phase	Enrolment	Study Designs	Start Date	Completion Date
**N-acetylcysteine**									
NCT04573153 (present Study)	Metabolic Cofactor Supplementation and Hydroxychloroquine Combination in Covid-19 Patients	Recruiting	Hydroxychloroquine + Metabolic Activators (N-acetylcysteine, Carnitine, Serine, Nicotinamide Riboside) Hydroxychloroquine + Sorbitol	18 and older	Phase 2|3	400	Randomized Open Label | Double Blinded Treatment	Sep-20	Mar-21
NCT04792021	Effect of N-acetylcysteine on Oxidative Stress in COVID-19 Patients	Recruiting	N-acetylcysteine	18 and older	Phase 3	60	Randomized Double blinded Treatment	Mar-21	Nov-21
NCT04755972	Mucolytics in Patients on Invasive Mechanical Ventilation Due to Severe Acute Respiratory Syndrome Coronavirus 2	Recruiting	N-acetylcysteine 5% sodium chloride 8,4% sodium bicarbonate	18 and older	Not Applicable	40	Randomized Open Label Prevention	Jan-21	Aug-21
NCT04466657	Antioxidant Therapy for COVID-19 Study	Not yet recruiting	Antioxidation Therapy	18 to 75	Not Applicable	90	Randomized Single blinded Supportive	Nov-20	Apr-21
NCT04703036	Glutathione, Oxidative Stress and Mitochondrial Function in COVID-19	Recruiting	Glycine N-acetylcysteine Alanine	55 to 85	Early Phase 1	64	Randomized Double blinded	Jan-21	Dec-21
NCT04279197	Treatment of Pulmonary Fibrosis Due to 2019-nCoV Pneumonia With Fuzheng Huayu	Recruiting	N-acetylcysteine+ Fuzheng Huayu Tablet	18 to 65	Phase 2	136	Randomized Double blinded treatment	Feb-20	Dec-22
NCT04370288	Clinical Application of Methylene Blue for Treatment of Covid-19 Patients	Recruiting	N-acetylcysteine Vitamin C Methylene blue	18 to 90	Phase 1	20	Randomized Open Label Treatment	Apr-20	Sep-20
NCT01962961	N-acetylcysteine to Reduce Oxidative Stress and Improve Endothelial Function in HIV-infected Older Adults	Completed	N-acetylcysteine	50 and older	Phase 1|2	24	Randomized Quadruple blinded Treatment	Oct-13	Oct-15
NCT03900988	Intravenous N-acetylcysteine and Oseltamivir Versus Oseltamivir in Adults Hospitalized With Influenza and Pneumonia	Not yet recruiting	N-acetyl cysteine	18 and older	Phase 4	160	Randomized Triple blinded Treatment	Apr-20	Jul-22

NCT03281226	RIPE vs RIPE Plus N-acetylcysteine in Patients With HIV/TB Co-infection	Recruiting	RIPE (2m) and RI (4m) RIPE+NAC (2m) and RI (4m)	18 and older	Phase 2	50	Randomized Open Label Treatment	Dec-16	Dec-19
NCT03982537	Effect of N-Acetyl Cysteine (NAC) on the Oral Microbiome	Not yet recruiting	N-Acetyl-L-Cysteine	18 and older	Phase 2	40	Randomize Open Label Supportive Care	May-20	Oct-23
NCT03967665	Risk Stratification-directed NAC for Prevention of Poor Hematopoietic Reconstitution	Recruiting	N-acetyl-L-cysteine	15 to 60	Phase 3	138	Randomized Open Label Prevention	Oct-18	Oct-24
NCT02348775	Glutathione and Function in HIV Patients	Active, not recruiting	N-acetylcysteine and glycine	45 to 65	Phase 1	16	Open Label	Nov-14	Aug-20
NCT01355198	Role of HIV on Glutathione Synthesis and Oxidative Stress	Completed	N-acetylcysteine and glycine	21 to 70	Phase 1	10	Open Label Treatment	Aug-10	Sep-11
NCT02930031	Redox Status and Immune Function	Completed	N-acetylcysteine Placebo	18 to 30	Not Applicable	10	Randomized Double blinded	Jan-15	Mar-16
NCT02080182	Effect of Acetylcysteine in Pediatric Acute Pyelonephritis.	Completed	Acetylcysteine	1 to 16	Phase 2	70	Randomized Quadruple blinded Treatment	Jan-14	Apr-16
NCT03460808	The Combination of Atorvastatin, Acetylcysteine and Danazol as the Treatment of Steroid-resistant/Relapse Immune Thrombocytopenia	Not yet recruiting	Atorvastatin Acetylcysteine Danazol	18 and older	Phase 1| 2	200	Open Label Treatment	Mar-18	Jan-23
NCT04368598	The Combination of High-dose Dexamethasone and Acetylcysteine as the Treatment of Newly-diagnosed ITP	Recruiting	Dexamethasone Acetylcysteine	18 to 80	Phase 2	44	Open Label Treatment	Apr-19	Dec-20
NCT04545008	Trial of Famotidine & N-Acetyl Cysteine for Outpatients With COVID-19	Not yet recruiting	Famotidine N-Acetyl cysteine	18 and older	Phase 1	42	Randomized Open Label Treatment	Sep-20	Aug-21
NCT02688361	A Bioequivalence Study of an Acetylcysteine 2% Oral Solution Versus a Reference Fluimucil 2% Oral Solution	Completed	Fluimucil 2% solution Acetylcysteine 2% solution	18 to 45	Phase 1	46	Randomized Open Label	Feb-16	Apr-16
NCT03069300	N-ACetylcysteine to Reduce Infection and Mortality for Alcoholic Hepatitis	Recruiting	N-acetyl cysteine	18 and older	Phase 3	42	Randomized Open Label Treatment	Oct-15	Jun-25
NCT04455243	Inflammatory Regulation Effect of NAC on COVID-19 Treatment	Not yet recruiting	N-Acetyl cysteine	18 and older	Phase 3	1180	Randomized Quadruple blinded Treatment	Aug-20	Aug-21
NCT03236220	Effect of NAC on the Hematopoietic Reconstitution After Haploidentical Hematopoietic Stem Cell Transplantation	Completed	N-acetyl-L-cysteine	15 to 60	Phase 2	35	Open Label Treatment	Aug-17	Dec-18
NCT04419025	Efficacy of N-Acetylcysteine (NAC) in Preventing COVID-19 From Progressing to Severe Disease	Not yet recruiting	N-acetylcysteine	18 and older	Phase 4	200	Randomized Open Label Treatment	Aug-20	May-21
NCT03197103	The Impact of N-Acetylcysteine on Volumetric Retention of Autologous Fat Graft for Breast Asymmetry Correction	Unknown status	N-Acetylcysteine	18 to 40	Phase 4	15	Randomized Quadruple blinded Treatment	Jul-17	May-18
NCT00650091	Evaluating the Effectiveness of Prednisone, Azathioprine, and N-acetylcysteine in Patients With IPF	Completed	N-acetylcysteine	35 to 85	Phase 3	264	Randomized Quadruple blinded Treatment	Oct-09	Jan-14
NCT02249546	Efficacy of Acetylcysteine-containing Triple Therapy in the First Line of Helicobacter Pylori Infection	Unknown status	N-acetylcysteine + PPI-amoxicillin-clarithromycin PPI-amoxicillin-clarithromycin	20 and older	Phase 4	654	Randomized Open Label Treatment	Sep-14	Oct-16
NCT00493610	Mucomyst for Hepatitis C	Suspended	N-acetylcysteine	18 to 80	Not Applicable	5	Non-Randomized Open Label Treatment	Nov-06	Jun-08
NCT01138956	Immune Response of Visceral Leishmaniasis Patients Treated With Antimonial Plus N-Acetylcysteine	Unknown status	N-acetylcysteine + Pentavalent antimonial	2 to 50	Not Applicable	40	Randomized Single blinded	Apr-10	Dec-11
NCT00962442	N-Acetylcysteine in Severe Acute Alcoholic Hepatitis	Completed	N-Acetylcysteine	18 and older	Phase 3	44	Randomized Open Label Treatment	Sep-00	-
NCT00775476	Treatment of Systemic Lupus Erythematosus (SLE) With N-acetylcysteine	Not yet recruiting	N-acetylcysteine	18 and older	Phase 2	290	Randomized Quadruple blinded Treatment	Oct-20	Jun-26

NCT04374461	A Study of N-acetylcysteine in Patients With COVID-19 Infection	Recruiting	N-acetylcysteine	18 and older	Phase 2	86	Non-Randomized Open Label Treatment	May-20	May-21
NCT04458298	A Study to Evaluate OP-101 (Dendrimer N-acetyl-cysteine) in Severe Coronavirus Disease 2019 (COVID-19) Patients	Recruiting	OP-101	18 and older	Phase 2	24	Randomized Double blinded Treatment	Jul-20	Nov-20
NCT00397735	N-acetylcysteine in Intra-amniotic Infection/Inflammation	Completed	Amniocentesis<br>N-acetylcysteine	18 and older	Phase 1| 2	68	Randomized Quadruple blinded Treatment	Oct-06	Aug-18
**L-Carnitine**									
NCT03604016	Study to Assess Efficacy of Besifovir and L-carnitine in Chronic Hepatitis B Patients With Nonalcoholic Fatty Liver	Not yet recruiting	Besifovir dipivoxil<br>L-carnitine<br>Tenofovir Alafenamide	20 and older	Phase 4	76	Randomized Open Label Treatment	Sep-18	Jul-20
NCT02312414	Effects of Carnitine on Oxidative Stress to IVIR Administration to CKD Patients:Impact of Haptoglobin Genotype	Unknown status	L-Carnitine	18 to 80	Not Applicable	25	Randomized Open Label Treatment	Oct-14	-
NCT01909557	Acetyl-L-Carnitine Supplementation During HCV Therapy With Peg IFN-Î±2b Plus Ribavirin: Effect on Work Performance.	Completed	Acetyl-L-carnitine	18 to 90	Phase 3	62	Randomized Double blinded Treatment	Jan-10	Dec-11
NCT01913964	Acetyl-L-Carnitine Supplementation During HCV Therapy With Pegylated Interferon-Î±2b Plus Ribavirin	Completed	Acetylcarnitine	45 to 65	Phase 4	-	Randomized Double blinded Treatment	Oct-97	Oct-97
NCT00225160	ALCAR Prophylaxis Study	Unknown status	Acetyl L-carnitine	18 and older	Phase 2	50	Randomized Double blinded Prevention	Nov-03	-
NCT00202228	Lactate Metabolism Study in HIV Infected Persons	Completed	Cofactor supplementation (thiamine, riboflavin, L-carnitine)	18 and older	Phase 4	30	Non-Randomize, Open Label Treatment	Jul-02	Sep-11
NCT00386971	Effects of L-Carnitine on Postprandial Clearance of Triglyceride-rich Lipoproteins in HIV Patients on HAART	Completed	L-carnitine	18 to 70	Not Applicable	13	Randomized Double blinded Treatment	Oct-06	Dec-09
NCT00572429	Effects of Mixed Exercise Regime and L-Carnitine Supplementation in HIV Patients on HAART	Withdrawn	L-carnitine	18 to 70	Not Applicable	0	Randomized Double blinded Treatment	Jul-08	Dec-10
NCT00050271	Acetyl-L-Carnitine for the Treatment of NRTI-Associated Peripheral Neuropathy	Completed	Acetyl-L-carnitine	13 and older	Not Applicable	27	Randomized Open Label Treatment	Jan-07	.
NCT00079599	L-Carnitine to Treat Fatigue in AIDS Patients	Completed	L-carnitine	18 and older	Phase 2	44	Randomized Quadruple blinded Treatment	Nov-02	Mar-07
**Nicotinamide riboside (a form of Vitamin B3, Niacin)**									
NCT04818216	Nicotinamide Riboside in SARS-CoV-2 (COVID-19) Patients for Renal Protection	Not yet recruiting	Nicotinamide riboside	18 and older	Phase 2	100	Randomized Double blinded Treatment	Apr-21	Apr-23
NCT00246376	Diet, Exercise, Niacin, and Fenofibrate to Reduce Heart Disease Risk Factors in Individuals with HIV Lipodystrophy or Dyslipidaemia	Completed	Diet<br>Exercise<br>Niacin<br>Fenofibrate	18 to 65	Not Applicable	221	Randomized Triple blinded Treatment	Jan-04	Feb-12
NCT00046267	Niacin for Treatment of Elevated Cholesterol and Triglycerides in HIV-Infected Patients	Completed	Niacin	18 and older	Not Applicable	30	Non-Randomized Open Label Treatment	-	-
NCT00152893	To Determine if Chromium Nicotinate Supplementation Will Improve Insulin Resistance in HIV Patients with Metabolic Abnormalities	Completed	Chromium nicotinate	18 and older	Phase 2	52	Randomized Quadruple blinded Prevention	Aug-02	Feb-08
NCT01683656	ER Niacin/Laropiprant Impact on Cardiovascular Markers and Atheroprogression in HIV-infected Individuals on cART	Terminated	Niacin/Laropiprant	40 and older	Phase 4	4	Randomized Quadruple blinded Treatment	Aug-12	Jul-14
NCT02018965	Niacin on Immune Activation: a Proof-of-concept Study	Completed	Niacin	18 and older	Phase 2	16	Randomized Open Label Treatment	Nov-11	Jun-17
NCT01426438	Endothelial Function, Lipoproteins, and Inflammation with Low HDL Cholesterol in HIV: ER Niacin Versus Fenofibrate	Completed	Niacin<br>Aspirin<br>Fenofibrate	18 and older	Phase 2	99	Randomized Open Label Treatment	Nov-11	Oct-13

NCT00986986	Study of Niacin on Endothelial Function in HIV-infected Subjects with Low High Density Lipoprotein Cholesterol Levels	Completed	Niacin	18 and older	Not Applicable	20	Randomized<br>Open Label<br>Treatment	Nov-07	Apr-10
NCT04271735	Pilot Study to Evaluate the Effect of Nicotinamide Riboside on Immune Activation in Psoriasis	Recruiting	Niacin	18 and older	Phase 2	40	Randomized<br>Triple blinded<br>Basic Science	May-20	Sep-23
NCT02812238	Study to Evaluate the Effect of Nicotinamide Riboside on Immunity	Completed	Nicotinamide riboside (NR)	18 and older	Not Applicable	38	Randomized<br>Quadruple blinded<br>Basic Science	Jun-16	Aug-18
NCT03962114	Effects of Vitamin B3 in Patients with Ataxia Telangiectasia	Enrolling by invitation	Vitamin B3	2 and older	Phase 2	24	N/A<br>Open Label<br>Treatment	Mar-19	Mar-20
NCT00170404	TB Nutrition, Immunology and Epidemiology	Completed	Folic Acid<br>Micronutrients: Vitamins B1, B2, B6, Niacin, B12, C, E.<br>Selenium<br>Vitamin A	18 to 65	Phase 3	887	Randomized<br>Double blinded<br>Treatment	Jun-00	Oct-05
NCT04407390	Effects of Nicotinamide Riboside on the Clinical Outcome of Covid-19 in the Elderly	Recruiting	Nicotinamide riboside<br>Placebo	70 and older	Phase 2	100	Randomized<br>Quadruple blinded<br>treatment	Jun-20	May-22

Table S2 Baseline demographics of the study population

Characteristics	Phase-2 Study			Phase-3 Study		
	**CMA group (n=71)**	**Placebo group (n=22)**	**Study cohort (n=93)**	**CMA group (n=229)**	**Placebo group (n=75)**	**Study cohort (n=304)**
**Age,** years	35.0 (19-66)	32.5 (20-58)	35.6 (19-66)	36.7 (18-63)	35.2 (18-66)	36.3 (18-66)
**Sex**						
Male	31 (44%)	6 (27%)	56 (60%)	136 (59%)	39 (52%)	175 (57.6%)
Female	40 (56%)	16 (73%)	37 (40%)	93 (41%)	36 (48%)	129 (42.4%)
**Underlying health conditions**						
Body-mass index, kg/m^2	24.9 (16.8-37.8)	24.7 (20.2-33.9)	24.8 (16.8–37.8)	27.0 (16.8-45.6)	25.8 (17.2-34.4)	26.7 (16.8–45.6)
Smoking	17 (24%)	6 (27%)	23(24.7%)	56 (24%)	21 (28%)	77 (25.3%)
Alcohol consumption	3 (4.2%)	2 (9.1%)	5(5.3%)	20 (8.7%)	9 (12%)	29 (9.5%)
Hypertension	2 (2.8%)	0	2 (2.1%)	25 (10.9%)	3 (4%)	28 (9.2%)
Diabetes Mellitus	5 (7.0%)	0	5 (5.3%)	15 (6.6%)	4 (5.3%)	19 (6.2%)
**Symptoms and signs**						
Fever	13 (18.3%)	6 (27.2%)	19 (20.4%)	68 (29.6%)	18 (24.0%)	86 (28.2%)
Cough	18 (25.4%)	11 (50.0%)	29 (31.1%)	102 (44.5%)	30 (40.0%)	132 (43.4%)
Breathing issues	4 (5.6%)	2 (9.1%)	6 (6.4%)	20 (8.7%)	8 (10.6%)	28 (9.2%)
Tiredness	35 (49.3%)	13 (59.1%)	48 (51.6%)	115 (50.2%)	38 (50.6%)	153 (50.3%)
Muscle or joint pain	42 (59.2%)	14 (63.6%)	56 (60.2%)	142 (62.0%)	43 (57.3%)	185 (60.8%)
Headache	33 (46.5%)	12 (54.5%)	45 (48.3%)	89 (38.8%)	27 (36.0%)	116 (38.1%)
Smell or taste disorder	19 (26.8%)	6 (27.3%)	25 (26.8%)	42 (18.3%)	21 (28.0%)	63 (20.7%)
Sore throat	19 (26.8%)	3 (13.6%)	22 (23.6%)	59 (25.7%)	14 (18.6%)	73 (24.0%)
Nausea or vomiting	5 (7.0%)	0	5 (5.3%)	18 (7.9%)	6 (8.0%)	24 (7.8%)

Diarrhea	2 (2.8%)	0	2 (2.1%)	6 (2.6%)	5 (6.6%)	11 (3.6%)

Values are n (%) or median (range).

Table S3. Summary of Univariate Cox regression analysis of overall treatment duration in Phase 2

	Phase 2 study			
	HR	Lower 95% CI	Upper 95% CI	P value
CMA	2.59	1.54	4.38	3.62E-04
HQ or FP	-	-	-	-
Age	1.01	0.99	1.03	2.02E-01
Gender	0.94	0.61	1.44	7.77E-01
Other treatment history	0.87	0.52	1.45	6.01E-01
Cigarette usage	1.29	0.80	2.08	3.05E-01
Alcohol usage	1.39	0.55	3.47	4.84E-01

Note: HR = hazard risk, CI = confidence interval. CMA: Combined Metabolic Activators; HQ: hydroxychloroquine; FP: Favipiravir

Table S4. Summary of Univariate Cox regression analysis of overall treatment duration in Phase 3

	Phase 3 study			
	HR	Lower 95% CI	Upper 95% CI	P value
CMA	5.63	4.11	7.71	<2e-16
HQ or FP	0.96	0.72	1.29	0.78
Age	1.00	0.99	1.02	0.427
Gender	1.33	1.05	1.67	0.0161
Other treatment history	1.10	0.98	1.23	0.0979
Cigarette usage	0.95	0.73	1.24	0.72
Alcohol usage	1.07	0.73	1.57	0.741

Note: HR = hazard risk, CI = confidence interval. CMA: Combined Metabolic Activators; HQ: hydroxychloroquine; FP: Favipiravir

SUPPLEMENTARY FIGURE LEGENDS

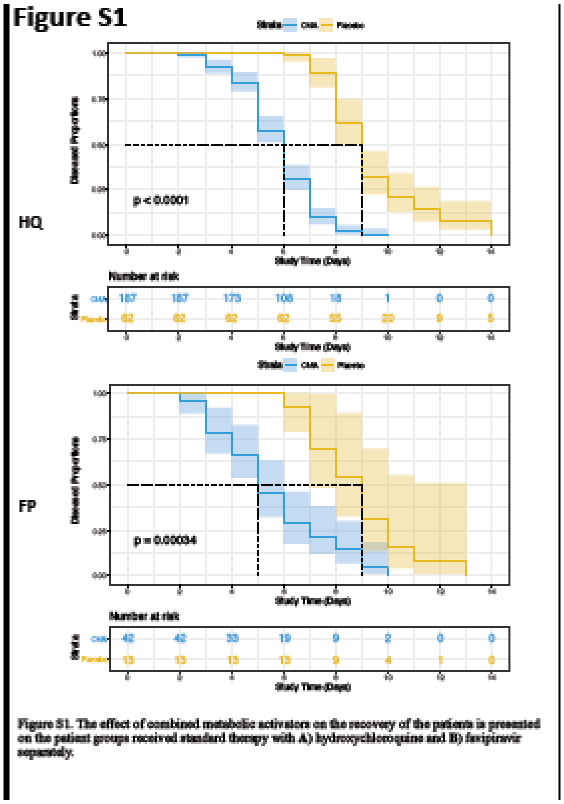

Figure S1. The effect of combined metabolic activators on the recovery of the patients is presented on the patient groups received standard therapy with **A)** hydroxychloroquine and **B)** favipiravir separately.

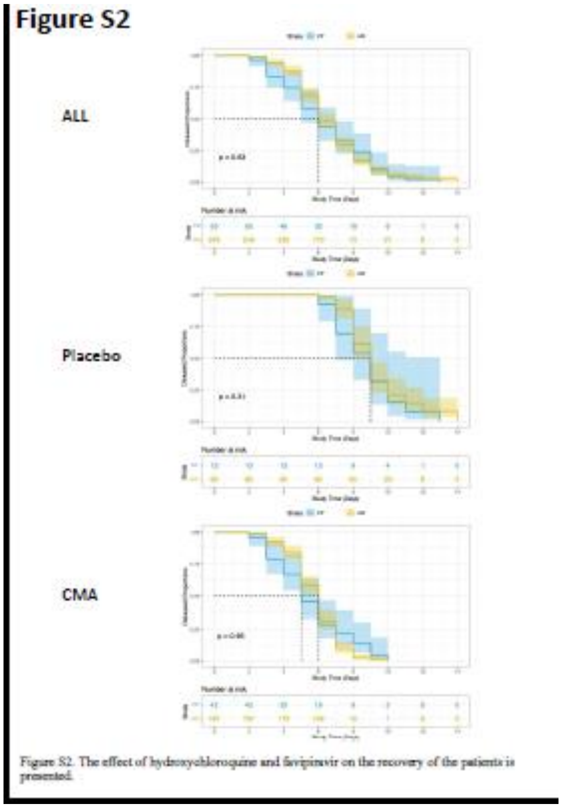

Figure S2. The effect of hydroxychloroquine and favipiravir on the recovery of the patients is presented.

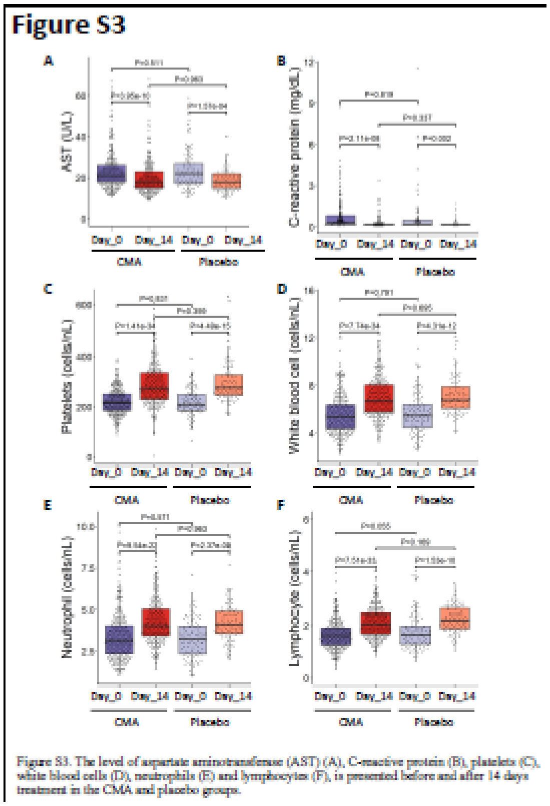

Figure S3. The level of aspartate aminotransferase (AST) **(A),** C-reactive protein **(B)**, platelets **(C)**, white blood cells **(D)**, neutrophils **(E)** and lymphocytes **(F)**, is presented before and after 14 days treatment in the CMA and placebo groups.

Figure S4

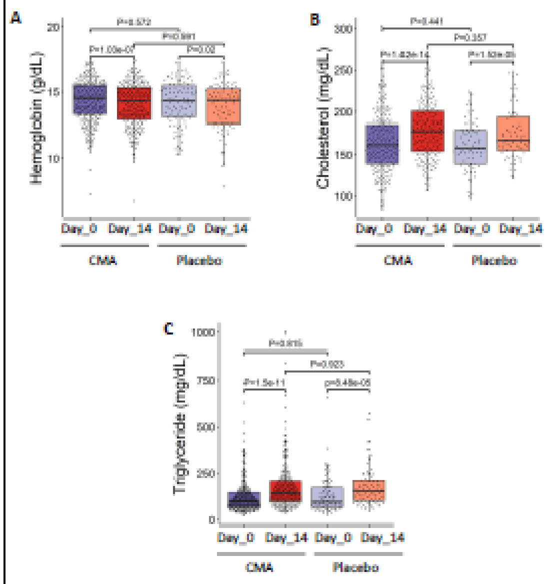

Figure S4. The level of the level of hemoglobin (A), cholesterol (B) and triglyceride (C) is presented before and after 14 days treatment in the CMA and placebo groups.

Figure S4. The level of the level of hemoglobin **(A)**, cholesterol **(B)** and triglyceride **(C)** is presented before and after 14 days treatment in the CMA and placebo groups.

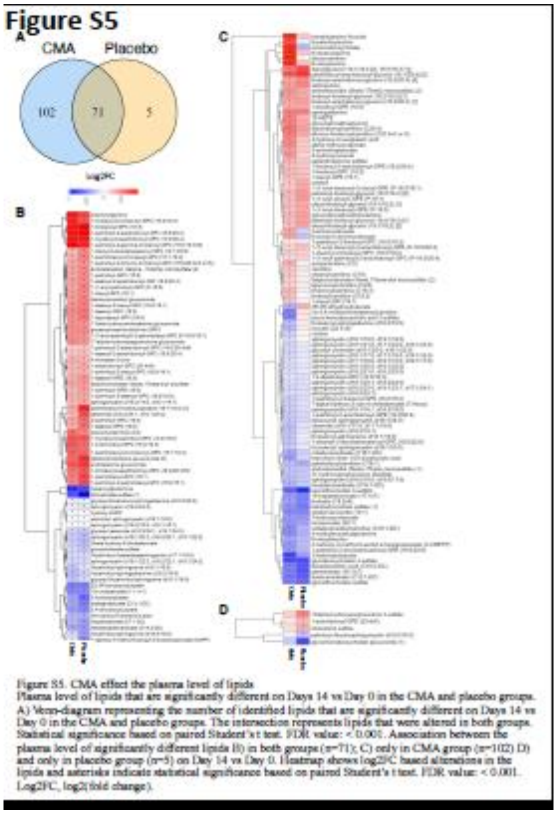

Figure S5. CMA effect the plasma level of lipids

Plasma level of lipids that are significantly different on Days 14 vs Day 0 in the CMA and placebo groups. **A)** Venn-diagram representing the number of identified lipids that are significantly different on Days 14 vs Day 0 in the CMA and placebo groups. The intersection represents lipids that were altered in both groups. Statistical significance based on paired Student's t test. FDR value: < 0.001. Association between the plasma level of significantly different lipids **B)** in both groups (n=71); **C)** only in CMA group (n=102) **D)** and only in placebo group (n=5) on Day 14 vs Day 0. Heatmap shows log2FC based alterations in the lipids and asterisks indicate statistical significance based on paired Student's t test. FDR value: < 0.001. Log2FC, log2(fold change).

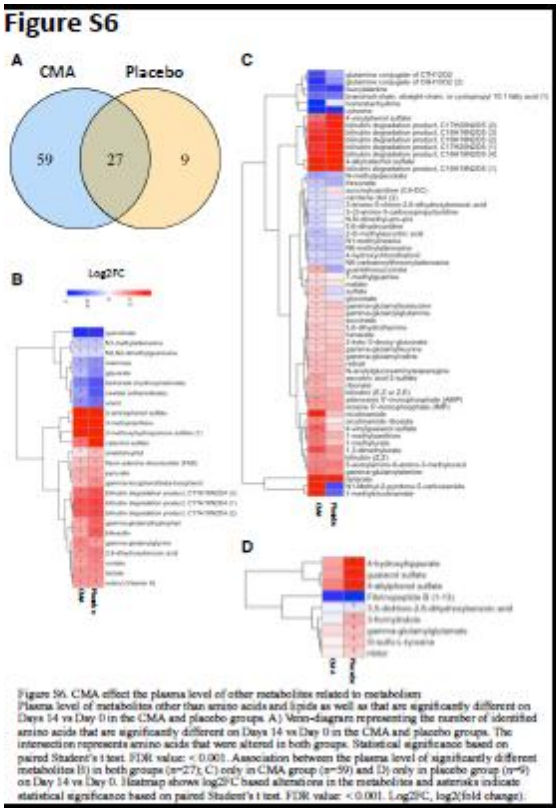

Figure S6. CMA effect the plasma level of other metabolites related to metabolism

Plasma level of metabolites other than amino acids and lipids as well as that are significantly different on Days 14 vs Day 0 in the CMA and placebo groups. **A)** Venn-diagram representing the number of identified amino acids that are significantly different on Days 14 vs Day 0 in the CMA and placebo groups. The intersection represents amino acids that were altered in both groups. Statistical significance based on paired Student's t test. FDR value: < 0.001. Association between the plasma level of significantly different metabolites **B)** in both groups (n=27); **C)** only in CMA group (n=59) and **D)** only in placebo group (n=9) on Day 14 vs Day 0. Heatmap shows log2FC based alterations in the metabolites and asterisks indicate statistical significance based on paired Student's t test. FDR value: < 0.001. Log2FC, log2(fold change).

SUPPLEMENTARY TABLES

Table S1 Clinical trials used N-acetyl-L-cysteine (NAC), Nicotinamide Riboside (NR) and L-carnitine in treatment of COVID-19 and other viral diseases.

Table S2 Baseline demographics of the study population

Table S3 Summary of univariate Cox regression analysis of overall treatment duration in the phase-2 study.

Table S4 Summary of univariate Cox regression analysis of overall treatment duration in the phase-3 study.

SUPPLEMENTARY DATASETS

Dataset S1 The characteristics of each patient involved in the Phase-2 and Phase-3 studies.

Dataset S2 Summary of laboratory and physical variables before and after treatment in the CMA and placebo groups in **A)** phase-2, **B)** phase-3 and **C)** all patients.

Dataset S3 Untargeted metabolomics data for each patient before and after treatment.

Dataset S4 Plasma level of metabolites that are significantly different on Days 14 vs Day 0 in the CMA and placebo groups. Only metabolites detected in >50% of samples were analyzed.

Dataset S5 Plasma level of metabolites that are significantly different between the CMA and placebo groups on Days 0 and Day 14. Only metabolites detected in >50% of samples were analyzed.

Dataset S6 Association between the plasma level all metabolites with the plasma levels of metabolic activators including serine, carnitine, nicotinamide riboside, and cysteine.

Dataset S7 The Olink multiplex inflammation panel used to detect the dynamic range of 72 proteins in plasma samples of the 93 patients participated in the study.

Dataset S8 Plasma level of inflammation related proteins that are significantly different on Days 14 vs Day 0 in the CMA and placebo groups. Only proteins detected in >50% of samples were analyzed.

Dataset S9 Plasma level of inflammation related proteins that are significantly different between the CMA and placebo groups on Days 0 and Day 14. Only metabolites detected in >50% of samples were analyzed.

Dataset S10 Association between the plasma level all inflammation related proteins with the plasma levels of metabolic activators including serine, carnitine, nicotinamide riboside, and cysteine.

Dataset S11 Association between the plasma level of clinical variables including ALT, AST, LDH, Triglycerides (TGs) and hemoglobin with plasma level of all metabolites.

Dataset S12 Association between the plasma level of clinical variables including ALT, AST, LDH, Triglycerides (TGs) and hemoglobin with plasma level of all inflammation related proteins.

Dataset S13 Association between the plasma level of key inflammation related proteins including MCP-4, IL-18R1, HGF, CXCL10 and CSF-1 with plasma level of all metabolites.
